# Cytotoxic Properties of Damiana (*Turnera diffusa*) Extracts and Constituents and A Validated Quantitative UHPLC-DAD Assay

**DOI:** 10.3390/molecules24050855

**Published:** 2019-02-28

**Authors:** Johanna Willer, Karin Jöhrer, Richard Greil, Christian Zidorn, Serhat Sezai Çiçek

**Affiliations:** 1 Department of Pharmaceutical Biology, Kiel University, Gutenbergstraße 76, 24118 Kiel, Germany; jwiller@pharmazie.uni-kiel.de (J.W.); czidorn@pharmazie.uni-kiel.de (C.Z.); 2 Tyrolean Cancer Research Institute, Innrain 66, 6020 Innsbruck, Austria; karin.joehrer@tkfi.at (K.J.); r.greil@salk.at (R.G.); 3 Department of Internal Medicine III, Oncologic Center, Salzburg Cancer Research Institute–Laboratory for Immunological and Molecular Cancer Research (SCRI-LIMCR), Paracelsus Medical University Salzburg, Cancer Cluster Salzburg, Müllner Hauptstraße 48, 5020 Salzburg, Austria

**Keywords:** multiple myeloma, quality control, naringenin, flavonoids, traditional preparation

## Abstract

In our continuing search for new cytotoxic agents, we assayed extracts, fractions, and pure compounds from damiana (*Turnera diffusa*) against multiple myeloma (NCI-H929, U266, and MM1S) cell lines. After a first liquid-liquid solvent extraction, the ethyl acetate layer of an acetone (70%) crude extract was identified as the most active fraction. Further separation of the active fraction led to the isolation of naringenin (**1**), three apigenin coumaroyl glucosides **2**–**4**, and five flavone aglycones **5**–**9**. Naringenin (**1**) and apigenin 7-*O*-(4″-*O*-*p*-*E*-coumaroyl)-glucoside (**4**) showed significant cytotoxic effects against the tested myeloma cell lines. Additionally, we established a validated ultra-high performance liquid chromatography diode array detector (UHPLC-DAD) method for the quantification of the isolated components in the herb and in traditional preparations of *T. diffusa*.

## 1. Introduction

*Turnera diffusa* Willd., Passifloraceae, commonly referred to as damiana, is a shrub occurring in north-eastern Brazil, Mesoamerica, the Caribbean, Mexico, and Texas [1]. The traditional use of *T. diffusa* in Latin America encompasses usage as an aphrodisiac, a tonic, and for the treatment of diabetes [2,3]. Damiana extracts with tequila are allegedly used as love potions [3]. Due to the long history of *T. diffusa* as an aphrodisiac, both the stimulating effect as well as the underlying mechanisms are relatively well investigated. In animal testing, the aqueous extract of *T. diffusa* was found to increase the sexual activity of rats [4]. The flavonoids obtained by percolation with methanol, and here especially the flavanone pinocembrin, were identified as aromatase inhibitors resulting in increased testosterone levels and improved libido [5]. Regarding the antidiabetic activity, conflicting results were obtained. Whereas Alarcon-Aguilar et al. could not find any hypoglycemic effect of an ethanol-water extract [6], Parra-Naranjo et al. demonstrated hypoglycemic effects of a methanol extract and identified teuhetenone A, a nor-sesquiterpene, as the active principle [7]. 

Apart from traditional usage, an aqueous extract of *T. diffusa* was found to inhibit the monoamine oxidase A with IC_50_ values of 130 mg/mL as well as the acetyl- and butyrylcholinesterase with IC_25_ values of 0.352 and 0.370 mg/mL, respectively [8]. Additionally, cytotoxic effects of a methanolic extract against breast carcinoma cell line MDA-MD-231 were demonstrated [9]. Though the mechanism behind the observed cytotoxicity remains unsolved, the compounds responsible for cell death induced by the methanolic extract were identified as arbutin and apigenin. 

The antioxidative effects of flavonoids are well documented and linked to certain structural features such as a dihydroxylated B-ring, a double bond located between C2 and C3, and a 4-oxo function at ring C [10]. Moreover, flavonoids are known to influence the metabolism, e.g., by inhibiting oxidases or activating antioxidative enzymes [11,12]. However, for flavonoids isolated from *T. diffusa* a variety of additional activities were reported. Velutin (**7**), a dimethoxylated hydroxyflavone known from the açaí berry (*Euterpe oleracea* Mart., Arecaceae), has been shown to possess strong anti-inflammatory effects by inhibiting NF-κB activation as well as p38 and JNK phosphorylation and hence by down-modulation of the expression of TNF-α and IL-6 [13]. At low doses, velutin (**7**) is more potent than established anti-inflammatory agents such as apigenin. Brito et al. found that the anti-inflammatory effect of velutin (**7**) against periodontitis is caused by an inhibition of HIF-1α expression [14]. Besides, the compound was reported to possess cytotoxic effects against human nasopharynx carcinoma (KB) cells with an IC_50_ value of 4.8 µM [15]. Acacetin 7-*O*-methyl ether (**9**), another methoxylated flavonoid, showed moderate cytotoxic effects on HeLa cells. Subsequent testing focused on the influence of aminoalkylation of acacetin 7-*O*-methyl ether (**9**) and the resulting antiproliferative activity against three human cancer cell lines (HeLa, HCC1954, SK-OV-3) [16]. The Mixe Indians (Oaxaca, Mexico) use an aqueous extract of *Calea zacatechichi* Schltdl. (Asteraceae) as a remedy for malaria. In subsequent experiments, the flavone genkwanin (**6**) was identified as active principle [17]. Boege et al. reported acacetin (**5**) to inhibit the topoisomerase I [18], though the compound lacks the structural feature (3-hydroxy group) supposed necessary for topoisomerase activity [19]. 

**Figure 1:**
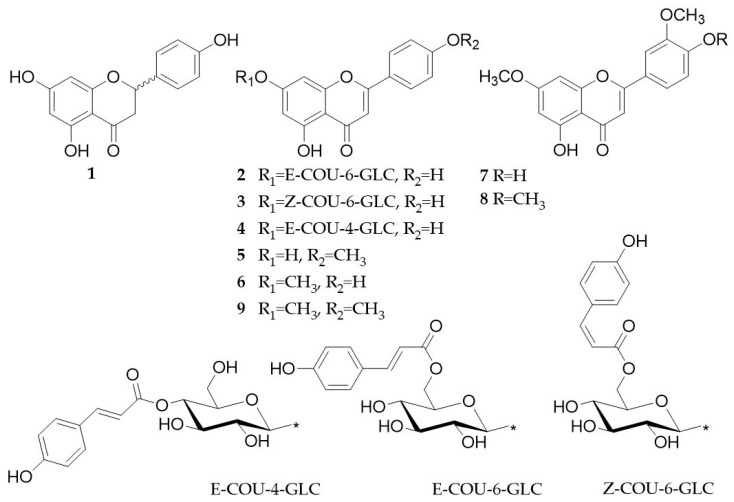
Chemical structures of flavonoids isolated from the aerial parts of *T. diffusa*. Naringenin (**1**), apigenin 7-*O*-(6″-*O*-*p*-*E*-coumaroyl)-glucoside (**2**), apigenin 7-*O*-(6″-*O*-*p*-*Z*-coumaroyl)-glucoside (**3**), apigenin 7-*O*-(4″-*O*-*p*-*E*-coumaroyl)-glucoside (**4**), acacetin (**5**), genkwanin (**6**), velutin (**7**), gonzalitosin I (**8**), and acacetin 7-*O*-methyl ether (**9**).

In the present study, the effect of extracts, fractions, and pure compounds from *T. diffusa* on multiple myeloma (MM) cell lines was investigated. Flavones, e.g., apigenin, chrysin, and luteolin, have been shown to block proteasome catalytic activities in tumor cells [20] and to induce cell death in myeloma cells [21]. Proteasome inhibitors are state-of-the-art in the therapy of multiple myeloma, but most patients develop resistance over time and new drugs are urgently needed. In addition, compounds of damiana are expected to induce reactive oxygen species [22], presumably adding to the expected cytotoxicity in myeloma cells. Our bioactivity-guided approach led to the isolation of seven flavonoids and a mixture of acacetin and genkwanin (Figure 1, **1**–**9**). In a second step, a validated ultra-high performance liquid chromatography (UHPLC) diode-array detector (DAD) method for the quantification of phenolic constituents in extracts and preparations of *T. diffusa* has been established.

## 2. Results

### 2.1. Bioactivity of Tested Fractions

**Figure 2:**
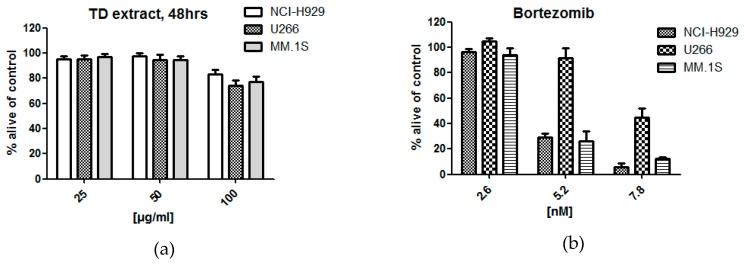

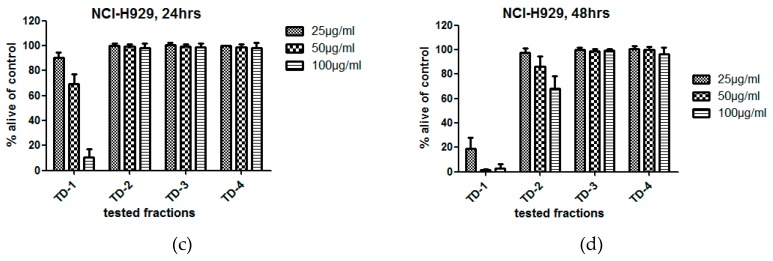
Viability of multiple myeloma (MM) cell lines NCI-H929, U266, and MM1S (**a**) 48 h after treatment with an acetone 70% extract of *T. diffusa* and after treatment with bortezomib (**b**). Viability of NCI-H929 cells 24 and 48 h after treatment with ethyl acetate (TD-1), *n*-butanol (TD-2), acidified *n*-butanol (TD-3) fractions and the remaining water layer (TD-4) derived from an acetone (70%) extract of *T. diffusa* (**c**,**d**). Three concentration levels (25, 50, 100 µg/mL) are shown.

After an acetone extract (70%) of *T. diffusa* showed cytotoxic potential in an initial screening against MM cell lines (Figure 2a,b (positive control)), the crude extract was fractionated with organic solvents of increasing polarity. Subsequently, the obtained fractions TD-1 (ethyl acetate), TD-2 (*n*-butanol), TD-3 (acidified *n*-butanol), and TD-4 (aqueous layer) were evaluated for their cytotoxicity. As shown in Figure 2c,d, the cytotoxic activity was mainly retained in the ethyl acetate fraction (TD-1), resulting in decreased viability in all tested concentrations after both 24 and 48 h of treatment, respectively. In contrast, treatment with TD-2 decreased the amount of viable cells only moderately after 48 h of treatment, whereas no effects were observed for fractions TD-3 and TD-4.

**Figure 3:**
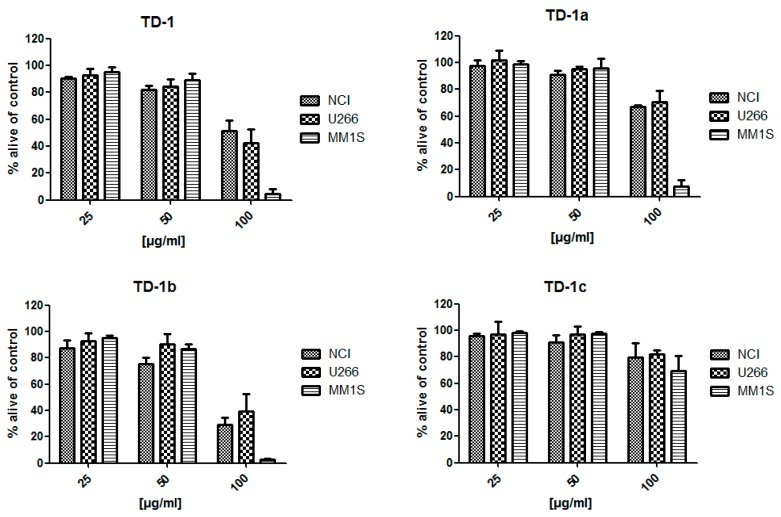
Viability of MM cell lines after treatment with fractions TD-1, TD-1a, TD-1b, and TD-1c. Three concentration levels (25, 50, 100 µg/mL) and 24 h incubation times are shown.

In a second approach, the crude acetone extract was fractionated in smaller polarity steps using *n*-hexane (TD-1a), diethyl ether (TD-1b), and ethyl acetate (TD-1c). The resulting fractions as well as TD-1 were then evaluated for their cytotoxic potential against MM cell lines MM1S, U266, and NCI-H929 after 24 h of incubation (Figure 3). As expected, TD-1 decreased viable cells significantly at a concentration of 100 µg/mL, but not at lower concentrations. Unlike before, partitioning in smaller polarity steps did not result in a single active fraction but revealed different activities for TD-1a to TD-1c. At a concentration of 100 µg/mL, TD-1a and TD-1b showed pronounced effects against MM1S cell lines, which were higher in TD-1b and lower in TD-1a, compared to TD-1. TD-1b moreover showed higher activity against NCI-H929 cell lines than TD-1 and slightly stronger effects against U266 cell lines. In contrast, TD-1a was less active against these two cell lines. TD-1c was only moderately active against all three tested cell lines.

### 2.2. Chromatographic Analyses of Tested Fractions

**Figure 4:**
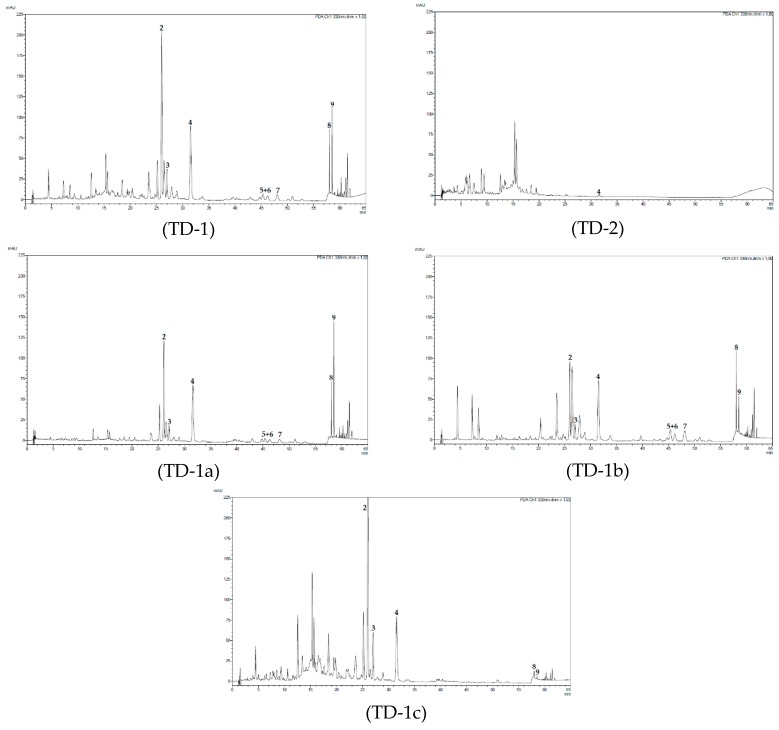
Ultra-high performance liquid chromatography (UHPLC) chromatograms of TD-1, TD-2, and TD-1a to TD-1c.

The tested fractions were analyzed by UHPLC in order to eventually attribute the observed effects to specific peaks (Figure 4). Comparison of fractions TD-1 and TD-2, which were obtained in the first separation step, clearly shows that the active fraction TD-1 contains a number of peaks that are missing in fraction TD-2. These peaks, which are eluting after 25 min comprise, amongst other, the later isolated compounds **2**, **3** and **5**–**9**. UHPLC analysis of the three fractions of the second partitioning step (TD-1a to TD-1c) resulted in similar findings. Here, fraction TD-1c, which was significantly less active than TD-1a and TD-1b, was lacking compounds **5**–**7** and showed clearly lower amounts of compounds **8** and **9**. However, compound **4** was present in more or less the same concentration and compounds **2**, **3** and various polar constituents were present in higher amounts. The difference between fraction TD-1a and the slightly more active fraction TD-1b was less significant. Apart from the presence of a few peaks in the more polar region, TD-1b showed higher amounts of compounds **5**–**8** and lower concentrations of compounds **2** and **9**. Thus, the isolation of peaks eluting after 25 min was considered most promising for the identification of the cytotoxic principle(s) present in *T. diffusa*. As the respective compounds were quantitatively extracted in the first separation step, but not in the second one, fraction TD-1 was chosen for isolation of the desired substances. 

### 2.3. Isolation of Natural Compounds

Separation of fraction TD-1 was accomplished by silica gel flash chromatography, Sephadex LH-20 gel permeation chromatography, as well as preparative medium- and semi-preparative high-performance liquid chromatography and yielded seven flavonoids and a mixture of two compounds. Using analytical reference standards, mass spectrometry (MS) and nuclear magnetic resonance (NMR) spectroscopy, the isolated compounds were identified as naringenin (**1**), apigenin 7-*O*-(6″-*O*-*p*-*E*-coumaroyl)-glucoside (**2**), apigenin 7-*O*-(6″-*O*-*p*-*Z*-coumaroyl)-glucoside (**3**), apigenin 7-*O*-(4″-*O*-*p*-*E*-coumaroyl)-glucoside (**4**), acacetin (**5**), genkwanin (**6**), velutin (**7**), gonzalitosin I (**8**), and acacetin 7-*O*-methyl ether (**9**).

### 2.4. Bioactivity of Isolated Compounds

**Figure 5:**
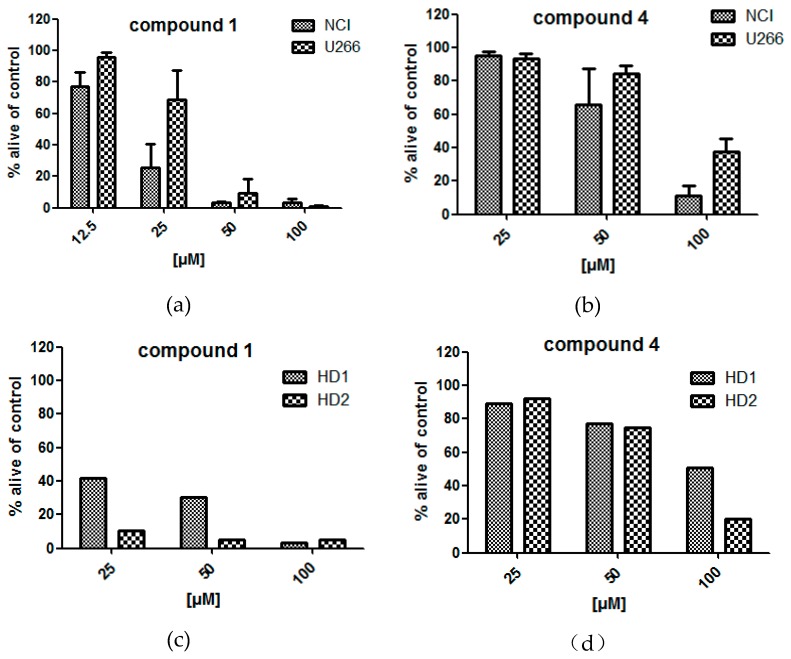
Viability of MM cell lines (U266, NCI-H929) (**a**,**b**) and healthy donor (HD) cells (**c**,**d**) 24 h after treatment with compounds **1** and **4**, respectively.

Cytotoxic assays of the pure compounds resulted in the identification of two compounds (**1** and **4**) with significant impact on cell viability (Figure 5). Of these two compounds, naringenin (**1**) was found cytotoxic even at low concentrations, showing a decreased viability of NCI-H929 and U266 cell lines of 25.5 ± 12.5 and 79.6 ± 15.2%, respectively, after 24 h incubation time. At the same incubation time and at a concentration of 50 µM, compound **4** decreased the viability of the same cell lines (NCI-H929 and U266) to 66.1 ± 17.4 and 84.4 ± 3.7%, respectively. Peripheral blood mononuclear cells (PBMC) from healthy donor (HD) were affected by the treatment with compounds **1** and **4**, if however, in different extent than the cancer cell lines. 

### 2.5. Validation of UHPLC-DAD Assay

**Figure 6:**
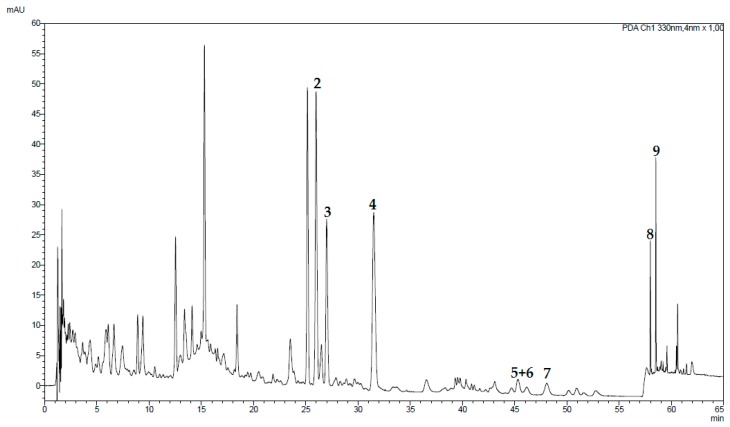
Chromatogram of a dimethyl sulfoxide (DMSO) extract prepared from *T. diffusa*. UHPLC was performed using a solvent mixture of 0.1% formic acid in water (solvent A) and acetonitrile (solvent B) with the following gradient: 15% B to 25% B in 15 min, to 29% B in 9 min, to 29% B in 11 min, to 36% B in 1 min, to 36% B in 19 min, to 95% B in 0.1 min, to 95% B in 9.9 min. Post-run was set to 10 min, temperature to 32 °C. The injection volume was 5 µL. The UV trace was recorded at 330 nm. The flow was 0.2 mL/min.

Of the isolated flavonoids, compounds **4** to **8** were chosen as calibration standards, using compound **4** to co-quantify compounds **2** and **3** and compound **8** to co-quantify compound **9**. Linearity was achieved by five-point calibration with a coefficient of determination of >0.99 (Table 1). Repeatability and precision measurements were performed using a dimethyl sulfoxide (DMSO) extract (Figure 6). Repeatability experiments revealed relative standard deviations (RSD) below 2.3% (Table 2). Intra-day precision showed RSD values below 6.9% for all compounds on day 1. The relative standard deviation on day 2 showed similar results for most compounds except for compounds **5**–**7**, which had higher deviations. Also, inter-day precision was higher for these compounds, with values of 6.9 and 7.8%, whereas the other compounds showed RSD values below 6%. Spiking experiments over four calibration levels conducted for compounds **4** to **8** showed recovery rates ranging from 98.0 to 127% (Table 3). 

### 2.6. Chromatographic Analyses of a Traditional Preparation

**Figure 7:**
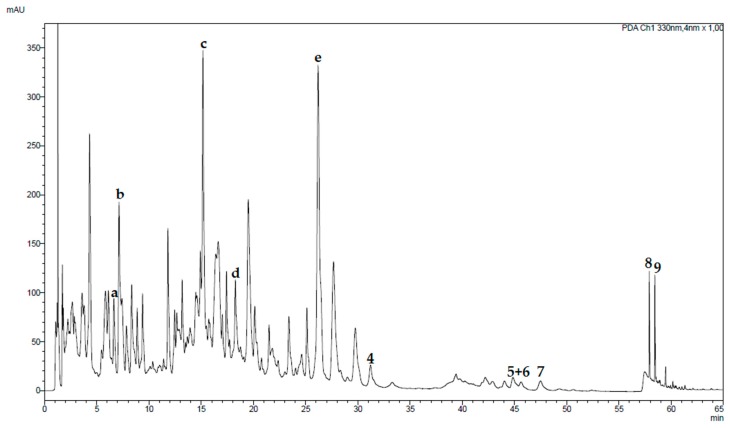
Chromatogram of a liquor prepared from *T. diffusa*. UHPLC was performed using a solvent mixture of 0.1% formic acid in water (solvent A) and acetonitrile (solvent B) with the following gradient: 15% B to 25% B in 15 min, to 29% B in 9 min, to 29% B in 11 min, to 36% B in 1 min, to 36% B in 19 min, to 95% B in 0.1 min, to 95% B in 9.9 min. Post-run was set to 10 min, temperature to 32 °C. The injection volume was 5 µL. The ultraviolet (UV) trace was recorded at 330 nm. The flow was 0.2 mL/min. **4** to **9** refer to the isolated compounds (see Figure 1), compounds **a** to **d** were identified by UV spectroscopy and MS spectrometry experiments as larycitrin-3-*O*-(6-glucosyl)glucoside (**a**), apigenin 8-*C*-(2-rhamnosyl)glucoside (**b**), (luteolin 8-*C*-(2-rhamnosyl)ketodeoxihexoside (**c**), apigenin 7-*O*-(2-rhamnosyl)ketodeoxihexoside (**d**), and **e** was identified as apigenin by an authentic standard.

The established method was additionally used for the analysis of a traditional preparation of *T. diffusa* (Figure 7). The sample of a traditional preparation of *T. diffusa* revealed minor compounds in the medium to low polarity range and a high content of polar compounds. By comparison with MS data and retention times of isolated compounds from *T. diffusa* compounds **4** to **9** were identified and their yield quantified using the method described above. Ultraviolet (UV) spectroscopy experiments indicated the presence of phenolic compounds in the polar range. Apigenin (**e**) was identified using reference standards. Previous investigations of an aqueous extract of *T. diffusa* by Bernardo et al. revealed a variety of apigenin, luteolin, and quercetin glycosides [8]. By comparison with ultraviolet (UV) and MS data larycitrin-3-*O*-(6-glucosyl)glucoside (**a**), apigenin 8-*C*-(2-rhamnosyl)glucoside (**b**), (luteolin 8-*C*-(2-rhamnosyl)ketodeoxihexoside (**c**), apigenin 7-*O*-(2-rhamnosyl)ketodeoxihexoside (**d**) were detected.

## 3. Discussion

### 3.1. Bioactivity of Tested Fractions

A crude acetone (70%) extract of *T. diffusa* displayed promising cytotoxicity against MM cell lines. Partitioning of the acetone extract located the cytotoxic compounds in the ethyl acetate (TD-1) fraction. In a second approach, using smaller polarity steps, the activity was found in the *n*-hexane (TD-1a) and the diethyl ether (TD-1b) fraction, while the remaining ethyl acetate fraction (TD-1c) was only slightly active. UHPLC experiments of the active fractions revealed similar peak patterns in the low polarity range. This peak pattern was absent in the inactive fraction TD-2, and different in TD-1c. Thus, further purification steps with fraction TD-1 focused on this polarity range and afforded the isolation of naringenin (**1**), apigenin 7-*O*-(6″-*O*-*p*-*E*-coumaroyl)-glucoside (**2**), apigenin 7-*O*-(6″-*O*-*p*-*Z*-coumaroyl)-glucoside (**3**), apigenin 7-*O*-(4″-*O*-*p*-*E*-coumaroyl)-glucoside (**4**), acacetin (**5**), genkwanin (**6**), velutin (**7**), gonzalitosin I (**8**), and acacetin 7-*O*-methyl ether (**9**).

Regarding the isolated compounds, compound **4** was abundant in all active fractions (TD-1, TD-1a to TD-1c) but was absent in TD-2. Treatment with compound **4** (50, 100 µM) led to decreased viability of NCI-H929 and in a lesser extent of U266 after incubation for 24 h. This indicates that compound **4** contributes to the cytotoxic effect observed for the tested fractions. The corresponding aglycone, the flavone apigenin, was reported repeatedly for its cytotoxic activity [9,20,21,23,24]. However, the moderately active fraction TD-1c contained less of lipophilic compounds **5** to **9**, suggesting that these compounds might at least contribute to the observed cytotoxic effects. 

The most active compound of our study, the flavanone naringenin (**1**), is well investigated for its ability to induce apoptosis against HL-60 cells via the activation of caspase-3, a member of the caspase-cascade that plays an important role in apoptosis. Interestingly, the cytotoxic effect of naringenin (**1**) was found to be weakened if C7 is substituted with a sugar moiety (rutinoside) [25,26]. In the present work, naringenin (**1**) was found to possess the highest activity of all tested substances and showed a pronounced decrease of viability especially in NCI-H929 cells. Nevertheless, the active principle resulting in the lowered viability in this study requires further testing. 

The experiments performed under the same conditions with HD cells indicate a negative influence on cells of the immune system after treatment with compound **1** or **4** (Figure 5). Interestingly, Chen et al. (2003) did not observe apoptosis in polymorphonuclear neutrophils (PMN) after their treatment with naringenin (**1**). However, testing conditions differed from the ones used in this study. Nevertheless, though flavonoids are generally assumed safe due to regular uptake with fruits and vegetables, they were found to evoke cytotoxic effects at higher doses [27,28].

### 3.2. Validation

By testing different columns (Synergi 4µm Polar-RP 80 Å 150 × 2.00 mm, Luna Omega 1.6 µm C18 100 Å 100 × 2.1 mm, Kinetex 1.7 µm XB-C18 100 Å 100 × 2.1 mm) the best separation was achieved for the C18 Luna Omega column. Consequently, it was chosen as starting point for method development. Different compositions of pure water or formic acid in water as well as methanol or acetonitrile were tested for their influence on the separation of the compounds of the DMSO extract. Thereby, 0.1% formic acid and acetonitrile were identified as suitable eluents and a column temperature of 32 °C was found to give a good resolution of the peaks. Due to the complex mixture and a variety of similar compounds within the crude extract, isocratic steps were performed at different solvent concentrations as part of the gradient. Because of the absorption maxima around 330 nm of compounds **4** to **8**, this wavelength was chosen for detection. An injection volume of 5 µL was found to provide a good repeatability with an acceptable peak resolution. 

The calibration curves for quantified constituents **4** to **8** were obtained on five levels each regarding the concentration of the corresponding analyte in the extract. The established calibration curves had determination coefficients of more than 0.99 and were thus accepted for quantification purposes. Spiking experiments showed acceptable recovery rates over a broad calibration range (Table 3).

Interday precision revealed good relative standard deviation (RSD) values for peaks **5** to **8**; compound **7** however varies in a bigger extent. These findings also account for the co-quantified peaks with acacetin 7-*O*-methylether (**9**) and the compounds **2** and **3** having similar deviations. Displaying structural isomers, the latter two compounds were co-quantified by compound **4**, while acacetin and genkwanin (**5** + **6**) were chosen to co-quantify acacetin 7-*O*-methylether (**9**) since the molecules only differ in the amount of one methyl group. 

Limit of quantification (LOQ) for all quantified compounds **4**–**8** was set to the lower limit of the calibration curve. Those values provide acceptable standard deviation (data not shown). Commonly, limit of detection (LOD) is defined as one third of LOQ. Therefore, LOD was calculated from the LOQ (Table 2). 

### 3.3. Traditional Preparation

*T. diffusa* is used as remedy for various diseases in the traditional medicine of Latin America. Additionally, a liquor of *T. diffusa* is used to increase sexual activity [3]. In literature, tequila is described as extraction agent while in this study Kornbrand (Bauerndank/Edeka/Hamburg, Germany) was used. Both liquors contain 38% ethanol and thus were considered comparable. Due to the contained water, the liquor was expected to contain a high number of polar compounds. This assumption was verified by UHPLC analysis, which revealed most of the peaks eluting in the polar range. Compounds **4** to **9** were present, however, at low concentrations. In the present study, compound **4** showed moderate cytotoxicity in an in vitro assay against myeloma cell lines and healthy donor (HD) cells at higher concentrations of 50 and 100 µM. This corresponds with 28.9 mg/mL or 57.8 mg/mL, a concentration unlikely to be met in traditional preparations.

## 4. Materials and Methods

### 4.1. General Experimental Procedures

Solvents and reagents for isolation were of analytical quality. Solvents used for UHPLC were of LC-MS grade quality. Column chromatography was performed with silica gel (40–63 μm particle size) (Merck, Darmstadt, Germany) or with Sephadex LH-20 (GE Healthcare, Chicago, IL, USA). Thin layer chromatography (TLC) was performed on silica gel 60 F_254_ plates (Merck) using ethyl acetate-methanol-water (10:1:1) or *n*-hexane-ethyl acetate-methanol-formic acid (7:4:1.5:0.1) as mobile phase and vanillin-sulphuric acid for detection. Preparative medium-performance liquid chromatography (MPLC) was accomplished using a Büchi PrepChrom C-700 equipped with a Büchi PrepChrom MPLC column C18 (250 × 30 mm, 15 µm) (Büchi, Flawil, Switzerland). Semi-preparative high-performance liquid chromatography (HPLC) was carried out on Waters a Alliance e2695 Separations Module with Alliance 2998 photodiode array (PDA), 2410 RI, and WFC III fraction collector (Waters, Milford, MA, USA), and either an Aqua 5 μ C18 column (250 × 10 mm, 5 µm particle size, Phenomenex, Aschaffenburg, Germany) or a VP Nucleodur C18 (250 × 10 mm, 5 µm particle size, Macherey-Nagel, Düren, Germany). Extracts, fractions, and pure compounds were analyzed by a Hitachi ChromasterUltra R_S_ System (VWR, Darmstadt, Germany) connected to an autosampler, column heater, PDA and a Sederé Sedex 100 evaporative light scattering detector (ELSD), using a Phenomenex Synergi Polar-RP column (150 × 2 mm, 4 µm particle size). Pure compounds were additionally analyzed by Nexera X2 system (Shimadzu, Kyoto, Japan) connected to an autosampler, column heater, PDA and a Shimadzu LCMS 8030 Triple Quadrupole Mass Spectrometer with electron spray ionization. Quantification was performed on the same instrument using a Phenomenex Luna Omega C18 column (100 × 2.1 mm, 1.6 µm particle size) and NMR spectra were recorded on an Avance III 300 NMR spectrometer (Bruker, Billerica, MA, USA) connected to a BACs-autosampler (Bruker). Centrifugation was performed on a Heraeus Megafuge 16 (Thermo Fisher Scientific Inc., Waltham, MA, USA). The authentic standard of Apigenin >98% HPLC was purchased from TransMIT (Gießen, Germany). 

### 4.2. Plant Material

Dried and cut aerial parts of *T. diffusa* (HAB-2014 quality) were obtained from Caesar & Loretz GmbH (Caelo), Hilden, Germany (art.-No.: 257a, lot number: 15294206). 

### 4.3. Extraction and Fractionation

Dried herb (1.00 kg) was ground and extracted five times with 2 liters of acetone 70% undergoing sonification. The solvent was evaporated under reduced pressure to afford 85 g of crude extract. For isolation the crude extract was repeatedly partitioned between ethyl acetate and water and the ethyl acetate layer was evaporated to dryness, yielding 32.6 g (TD-1). Subsequently, this procedure was repeated with butanol (14.6 g, TD-2). After acidification of the water layer with 2.5 mL formic acid, the solution was again extracted with butanol (5.40 g, TD-3) and the aqueous layer was then evaporated to dryness, yielding 32.4 g (TD-4). 

In a second approach, 5.0 g of crude extract were suspended in water and extracted with *n*-hexane. The fraction was evaporated to dryness, yielding 0.53 g (TD-1a). The procedure was repeated with diethyl ether (0.21 g, TD-1b) and ethyl acetate (0.22 g, TD-1c). 

For the validated UHPLC-DAD assay, sieved plant material (800 µm mesh width) was extracted threefold with DMSO, centrifuged, and the supernatants were collected and diluted in a 20 mL volumetric flask. 

The traditional liquor from *T. diffusa* was prepared by maceration of 35 g of ground drug material with 0.7 liters of 38% alcohol (Bauerndank) for four weeks. Subsequently, the extract was filtered.

### 4.4. Isolation

TD-1 was subjected to silica gel column chromatography (40 × 8 cm) and eluted in a gradient manner with petroleum ether-ethyl acetate-methanol-water (10:0:0:0 to 0:0:19:10) yielding 160 fractions. After characterization by TLC the obtained subfractions were combined to 20 fractions (TD-1_1 to TD-1_20).

From TD-1_2 compound **1**, a two to one mixture of **5** and **6**, and compound **8** were obtained after column chromatography with Sephadex LH20 and acetone–dichloromethane (15:85) as eluting solvent. The thereby eluted subfraction TD-1_2_D was subjected to semi-preparative HPLC using 0.025% formic acid in water and methanol in a gradient matter, yielding 14.2 mg of velutin (**7**) and 27.8 mg of linoleic acid. 27 mg of acacetin 7-*O*-methylether (**9**) and another 58 mg of linoleic acid were obtained from TD-1_4 by preparative column chromatography using 0.025% formic acid in water and methanol in a gradient matter. TD-1_8 (196 mg) was submitted to further separation by Sephadex LH20 (using acetone as eluent) to give 11.7 mg of compound **4**. TD-1_12 was subjected to semi-preparative HPLC using 0.025% formic acid in water and acetonitrile in an isocratic matter, yielding 6.3 mg of compound **3**. TD-1_14 yielded 10.0 mg of sitosterol and TD-1_ 20 9.0 mg of compound **2**. Structure elucidation of the isolated compounds was accomplished by comparison of MS- and NMR-spectra with literature data [29,30,31,32,33,34,35,36]. MS and NMR spectra (^1^H, HSQC, HMBC) are provided in the Supplementary Materials (**S1** to **S15**). 

### 4.5. Cytotoxic Assays

Cytotoxicity was assessed for TD-1 to TD-4, TD-1a to TD-1c as well as compounds **1** and **3** to **9**. Induction of apoptosis was measured in myeloma cell lines NCI-H929, MM1S, and U266 as well as in PBMCs of healthy donors by flow cytometry using established protocols [37] thereby staining the cells with Annexin-fluorescein isothiocyanate and propidium iodide. Bortezomib (Eubio, Vienna, Austria) was used as positive control. Cell lines were purchased from DSMZ (Braunschweig, Germany) and routinely fingerprinted and tested for mycoplasma negativity. All cells (cell lines and PBMC) were grown in RPMI-1640 medium (Life Technologies, Paisley, UK) supplemented with 10% fetal calf serum (FCS; PAA, Linz, Austria), l-glutamine 100 µg/ml, and penicillin-streptomycin 100 U/ml. PBMCs from healthy donors were utilized after obtaining written informed consent at the University Hospital Salzburg (ethics committee approval 415-E/1287/6-2011). Cells were subjected to Ficoll separation (Ficoll Paque^TM^, VWR, Darmstadt, Germany), and incubated in RPMI-1640 Media with supplements as above. In brief, 0.5 × 10^6^ myeloma cells/mL or similar numbers of PMBCs were incubated for 24 h and 48 h with or without the tested compounds dissolved in DMSO in different concentrations. At least three analysis in triplicates were performed for each cell line and a solvent control was included. The extent of non-apoptotic cells (AnnexinV/propidium iodide negative) was calculated as percentage of viable cells in respect to the untreated control. Data are shown as mean percentage of viable cells and standard deviation (error bars).

### 4.6. Chromatographic Analyses

Solvents used for UHPLC analyses during isolation steps were 0.1% formic acid in water and methanol using a gradient from 40% of methanol to 95% in 80 min with a flow of 0.2 mL/min. Post-run was 10 min, injection volume 5 µL, temperature 30 °C. UV traces were detected at 210 nm, 254 nm, and 280 nm. Additionally, an evaporative light scattering signal was recorded. 

For quantification a solvent mixture of 0.1% formic acid in water (solvent A) and acetonitrile (solvent B) was used with the following gradient: 15% B to 25% B in 15 min, to 29% B in 9 min, to 29% B in 11 min, to 36% B in 1 min, to 36% B in 19 min, to 95% B in 0.1 min, to 95% B in 9.9 min. Post-run was set to 10 min, temperature to 32 °C. The injection volume was 5 µL. The UV trace was recorded by at 330 nm. The flow was 0.2 mL/min. 

### 4.7. Method Validation

The UHPLC-DAD method was validated for linearity, LOD and LOQ, accuracy, precision, and repeatability. For the determination of linearity calibration curves were established by serial dilution of compounds **4** to **8**. Thus, calibration ranges of 5 µg/mL to 50 µg/mL (**4**), 0.75 µg/mL to 7.5 µg/mL (**7**), and 0.5 µg/mL to 5 µg/mL (**5** + **6** and **8**) were obtained. Corresponding regressions curves, coefficients of determination as well as LOD and LOQ are given in Table 1.

Repeatability was determined by measuring one sample six-fold while intra-day precision was studied by measuring six different samples once. Interday precision of the method was verified by assessing six samples on two different days. Consistency of compound concentrations was thereby investigated (Table 2).

Spiking experiments were performed on four levels for each quantified compound. For compound **5** + **6**, **7**, and **8** stock solutions of 0.04 mg/mL were prepared. From these, 0.75 mL, 0.5 mL or 0.25 mL were taken and mixed with 0.25 mL, 0.5 mL or 0.75 mL of the plant extract. Additionally, for all quantified constituents 0.25 mL of the lowest level of the calibration curve was added to 0.75 mL of the extract. Of these resulting solutions 5 µL were injected three-fold. Results are given in Table 3**.**

Acacetin 7-*O*-methylether (**9**) was co-quantified by acacetin and genkwanin (**5** + **6**), compounds **2** and **3** by compound **4**. 

## 5. Conclusions

Investigation of the cytotoxic properties of *T. diffusa* revealed significant effects for different apolar extracts against the myeloma cell lines MM1S, U266 and NCI-H929. Systematic evaluation of the active extracts by UHPLC led to the reduction of the complex metabolite to a range of possible candidates, which were subsequently isolated. Of these compounds, naringenin (**1**) and apigenin 7-*O*-(4″-*O*-*p*-*E*-coumaroyl)-glucoside (**4**) were identified as two components responsible for the observed activity. The cytotoxicity of naringenin is in line with previous findings, if however, observed for other cell lines [25,26]. Up to the best of our knowledge, compound **4** is described as cytotoxic for the first time. Nevertheless, its aglycone apigenin has been found active against cancer cell lines before [9,20,21,23,24]. Interestingly, only one of the two tested apigenin coumaroyl glucosides (compounds **3** and **4**) showed activity in the conducted assays, indicating steric effects to play a pivotal role for the cytotoxicity of these compounds.

Furthermore, the present study describes the first validated UHPLC-DAD method for the quantification of phenolic constituents in *T. diffusa*. The established assay allows the quantitation of eight flavonoids in both, the herb and the traditional preparation of *T. diffusa*, and coupled to mass spectrometry gives information on the abundance of another five flavonoids occurring in hydroethanolic damiana extracts.

## Figures and Tables

**Table 1:** Regression curves, coefficients of determination, limit of detection (LOD), and limit of quantitation (LOQ) of the UHPLC method.

Compound	Regression Curve	R^2^	LOD ^1^	LOQ ^1^
**4**	$6.971\times 10^{9}\times (-3.027\times 10^{5})$	0.9985	1.39	4.60
**5 + 6**	$8.170\times 10^{9}\times (-1.455\times 10^{6})$	0.9955	0.162	0.535
**7**	$3.605\times 10^{9}\times (-5.394\times 10^{5})$	0.9956	0.236	0.780
**8**	$9.720\times 10^{9}\times (-1.862\times 10^{6})$	0.9924	0.120	0.396

^1^ Concentrations are given in µg/mL.

**Table 2:** Repeatability and precision of the UHPLC method.

Compound	Repeatability ^1^	Intra-day 1 ^1^	Intra-day 2 ^1^	Inter-day ^1^
**2**	12.9 (0.138)	10.9 (4.95)	10.6 (4.90)	10.8 (4.82)
**3**	2.16 (2.29)	1.73 (5.52)	1.68 (4.20)	1.71 (4.97)
**4**	8.38 (1.19)	7.73 (4.18)	7.29 (4.83)	7.51 (5.25)
**5 + 6**	1.96 (1.62)	1.72 (3.02)	1.55 (5.48)	1.64 (6.89)
**7**	2.12 (1.05)	1.63 (6.89)	1.71 (9.01)	1.67 (7.82)
**8**	1.08 (0.389)	0.907 (3.37)	0.897 (3.66)	0.902 (3.40)
**9**	1.73 (0.169)	1.39 (4.87)	1.39 (5.34)	1.39 (4.88)

^1^ Concentrations are given in µg/mL, relative standard deviations are given in parentheses and are stated in percent.

**Table 3:** Accuracy of the UHPLC method.

Compound	Sample Concentration ^1^	Spiked Amount ^2^	Total Concentration ^1^	Recovery ^3^
**4**	6.56	3.08	4.72	105
		7.69	12.6	113
		15.4	18.7	112
		23.1	24.7	111
**5 + 6**	1.38	0.401	0.746	102
		1.07	2.10	99.7
		2.14	2.83	106
		3.21	3.55	107
**7**	1.52	0.585	0.964	102
		1.04	2.18	100
		2.08	2.84	102
		3.12	3.50	99.2
**8**	0.798	0.297	0.497	109
		0.793	1.39	109
		1.59	1.99	109
		2.09	2.29	127

^1^ Concentrations are given in µg/mL. ^2^ Amounts are given in µg. ^3^ Recovery is stated in percent.

## Supplementary Materials

The following are available online, Figures S1–S15.
