# Supplementary material for: Cytotoxic Properties of Damiana (Turnera diffusa) Extracts and Constituents and A Validated Quantitative UHPLC-DAD Assay
Source: Molecules. 2019 Feb 28;24(5):855. doi: 10.3390/molecules24050855 (PMC6429218; doi:10.3390/molecules24050855)
Supplement: Supplementary file 1 [file molecules-24-00855-s001.pdf]

Table of contents

Fig. S1. MS spectra of naringenin (**1**).

Fig. S2. ^{1}H-NMR- (**a**), HSQC- (**b**), HMBC- (**c**) spectra of apigenin 7-*O*-(6′′-*O*-*p*-*E*-coumaroyl)-glucoside (**2**).

Fig. S3. MS spectra of apigenin 7-*O*-(6′′-*O*-*p*-*E*-coumaroyl)-glucoside (**2**).

Fig. S4. ^{1}H-NMR- (**a**), HSQC- (**b**), HMBC- (**c**) spectra of apigenin 7-*O*-(6′′-*O*-*p*-*Z*-coumaroyl)-glucoside (**3**).

Fig. S5. MS spectra of apigenin 7-*O*-(6′′-*O*-*p*-*Z*-coumaroyl)-glucoside (**3**).

Fig. S6. ^{1}H-NMR- (**a**), HSQC- (**b**), HMBC- (**c**) spectra of apigenin 7-*O*-(4′′-*O*-*p*-*E*-coumaroyl)-glucoside (**4**).

Fig. S7. MS spectra of apigenin 7-*O*-(4′′-*O*-*p*-*E*-coumaroyl)-glucoside (**4**).

Fig. S8. ^{1}H-NMR- (**a**), HSQC- (**b**), HMBC- (**c**) spectra of acacetin and genkwanin (**5**+**6**).

Fig. S9. MS spectra of acacetin and genkwanin (**5**+**6**).

Fig. S10. ^{1}H-NMR- (**a**), HSQC- (**b**), HMBC- (**c**) spectra of velutin (**7**).

Fig. S11. MS spectra of velutin (**7**).

Fig. S12. ^{1}H-NMR- (**a**), HSQC- (**b**), HMBC- (**c**) spectra of gonzalitosin I (**8**).

Fig. S13. MS spectra of gonzalitosin I (**8**).

Fig. S14. ^{1}H-NMR- (**a**), HSQC- (**b**), HMBC- (**c**) spectra of acacetin 7-*O*-methyl ether (**9**).

Fig. S15. MS spectra of acacetin 7-*O*-methyl ether (**9**).

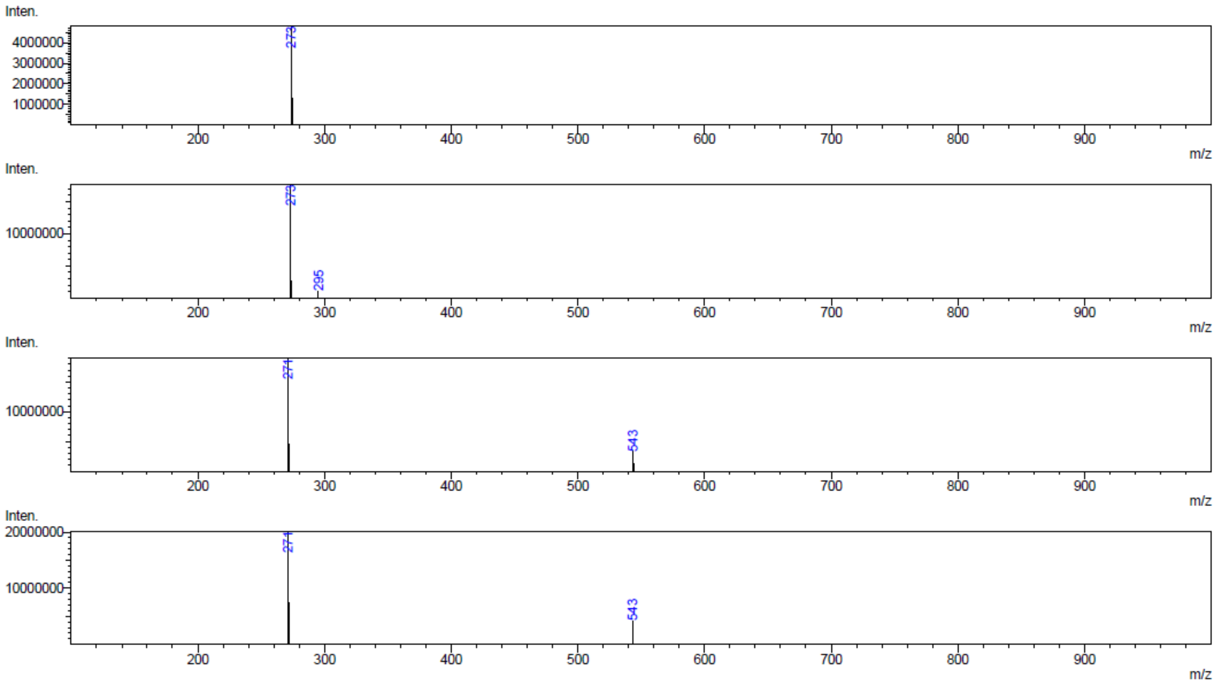

Fig. S1. ESI-MS spectra of naringenin (**1**). Spectra were recorded in positive ion mode (Q1+, Q3+) and negative ion mode (Q1-, Q3-).

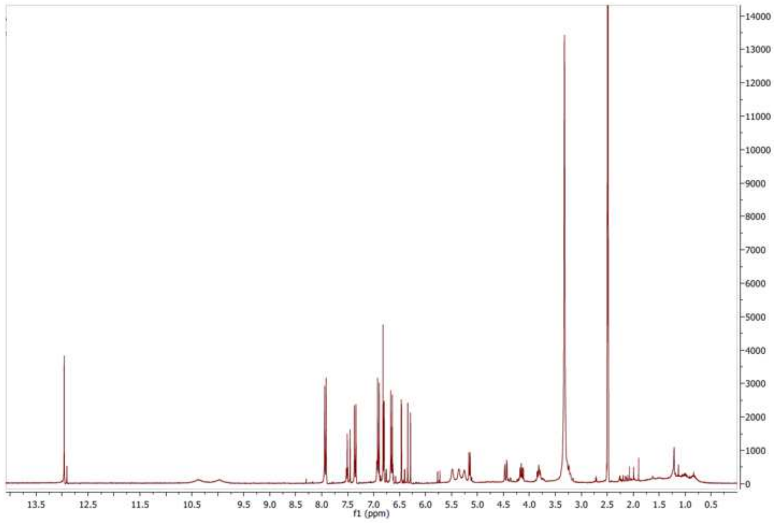

(a)

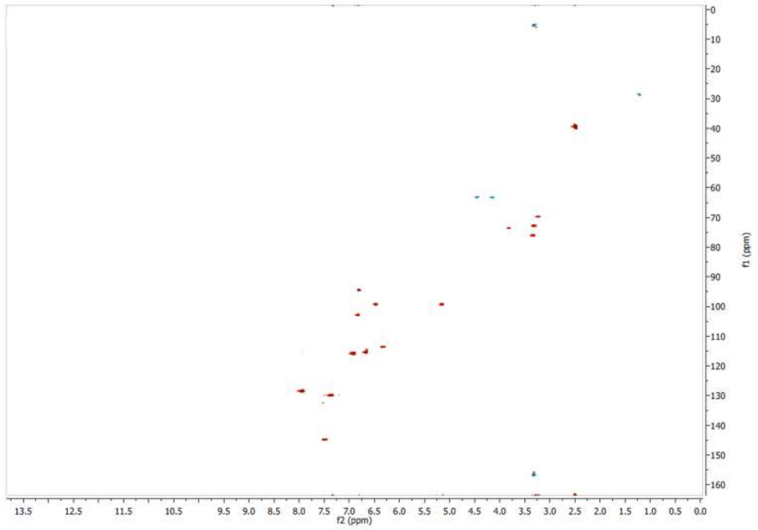

(b)

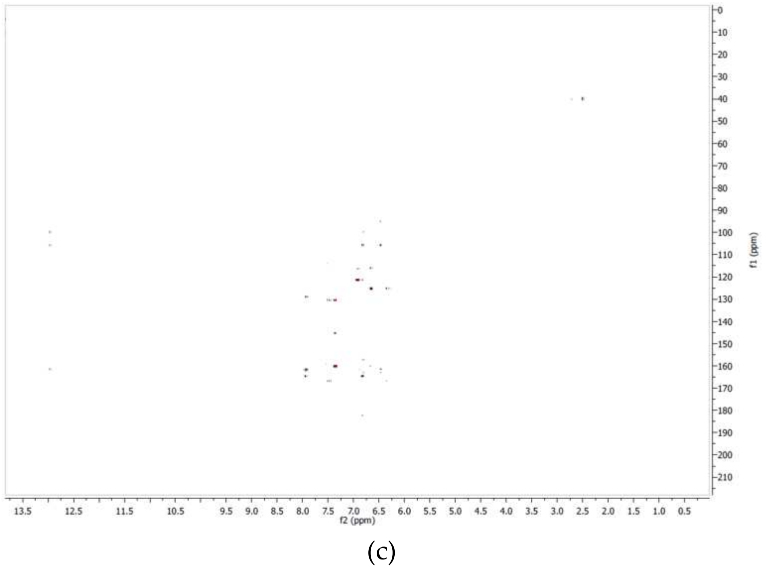

(c)

Fig. S2. ^{1}H-NMR- (**a**), HSQC- (**b**), HMBC- (**c**) spectra of apigenin 7-*O*-(6′′-*O*-*p*-*E*-coumaroyl)-glucoside (**2**).

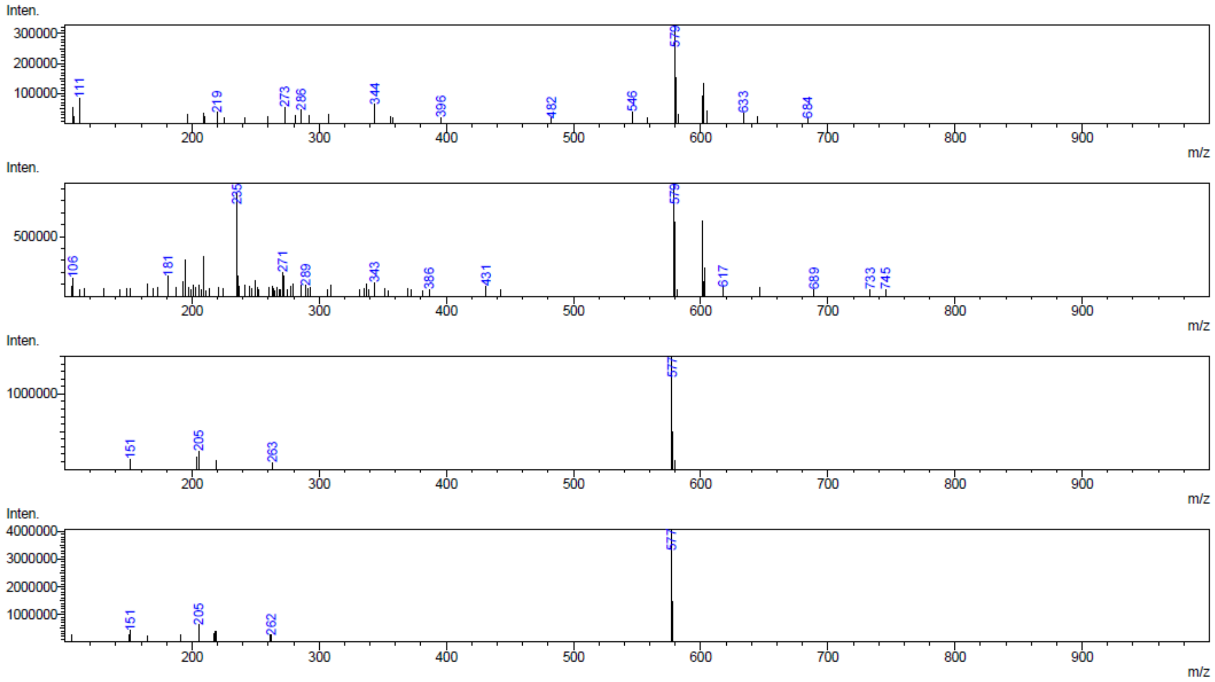

Fig. S3. ESI-MS spectra of apigenin 7-*O*-(6′′-*O*-*p*-*E*-coumaroyl)-glucoside (**2**). Spectra were recorded in positive ion mode (Q1+, Q3+) and negative ion mode (Q1-, Q3-).

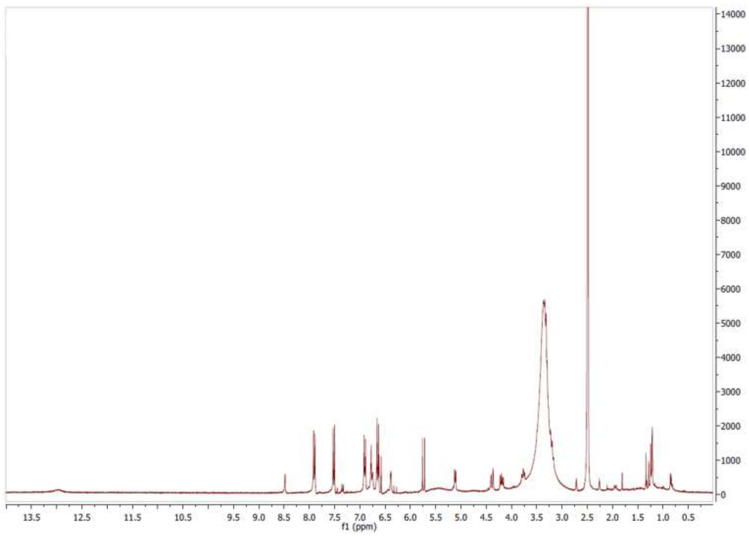

(a)

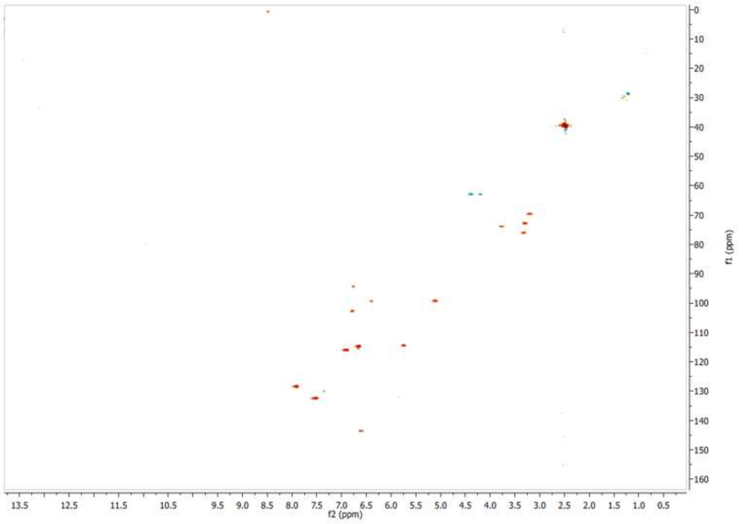

(b)

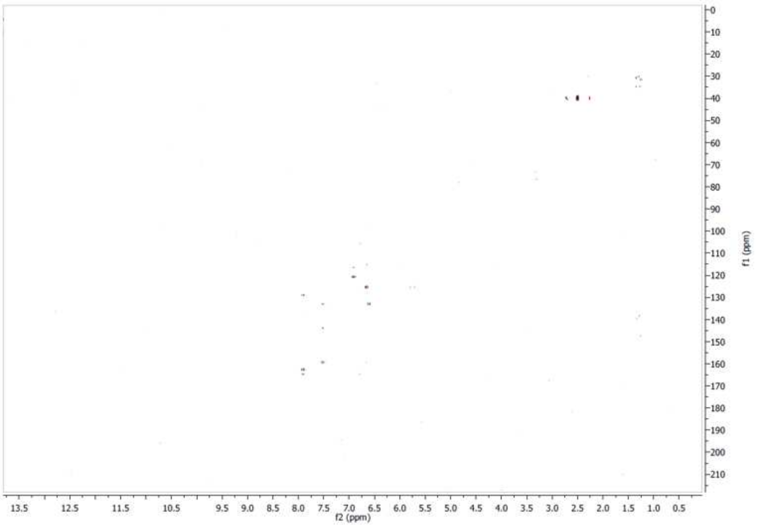

(c)

Fig. S4. ^{1}H-NMR- (**a**), HSQC- (**b**), HMBC- (**c**) spectra of apigenin 7-*O*-(6′′-*O*-*p*-*Z*-coumaroyl)-glucoside (**3**).

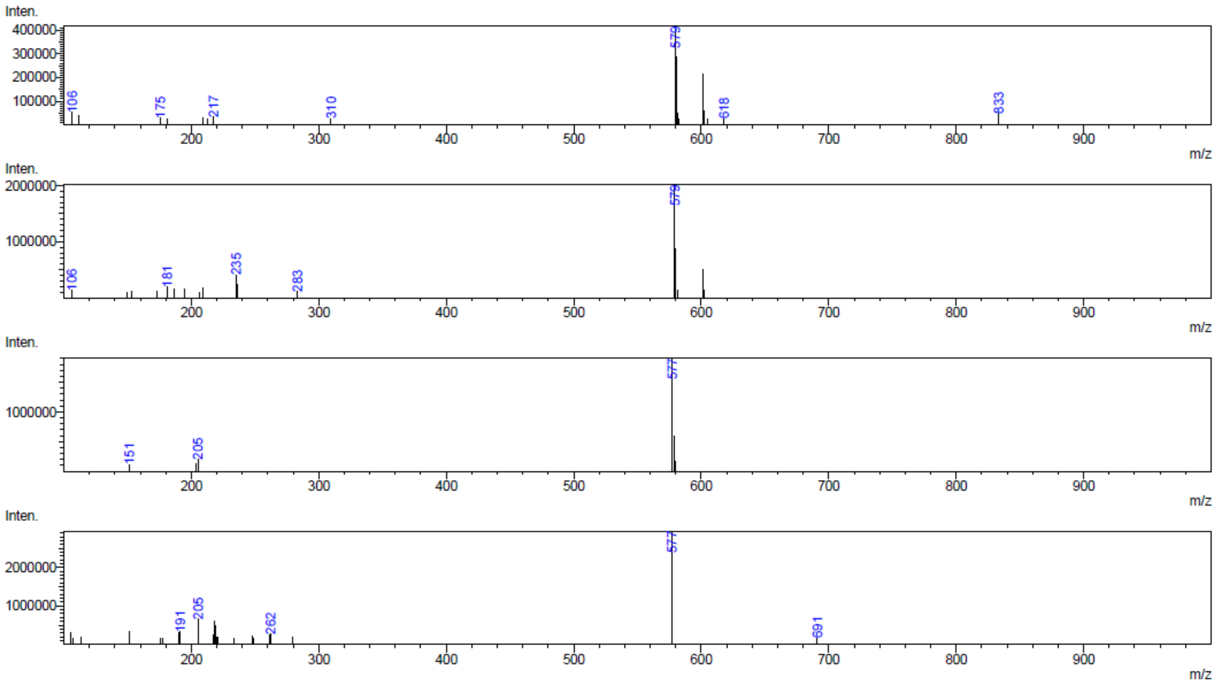

Fig. S5. ESI-MS spectra of apigenin 7-*O*-(6′′-*O*-*p*-*Z*-coumaroyl)-glucoside (**3**). Spectra were recorded in positive ion mode (Q1+, Q3+) and negative ion mode (Q1-, Q3-).

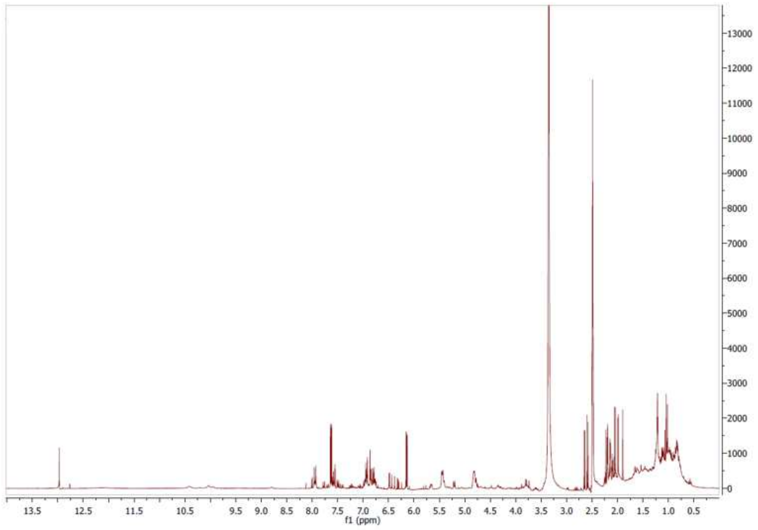

(a)

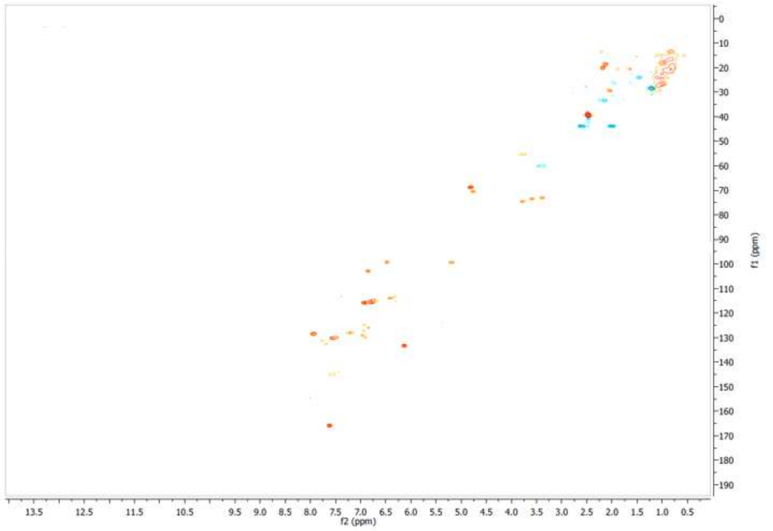

(b)

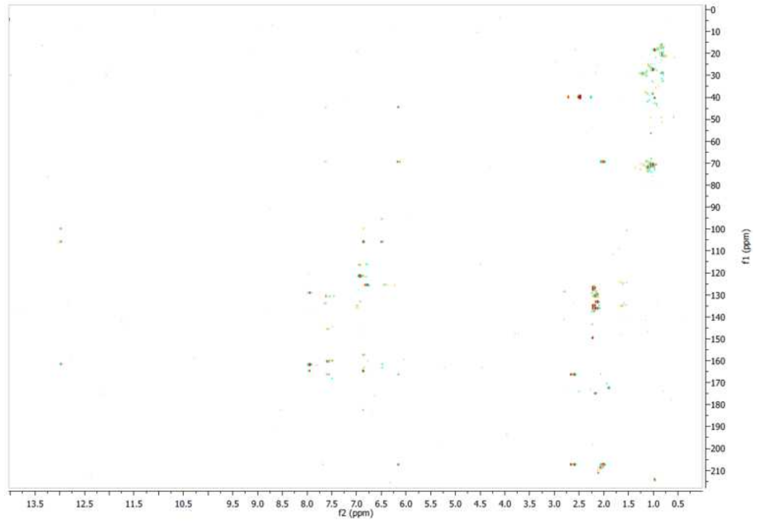

(c)

Fig. S6. ^{1}H-NMR- (**a**), HSQC- (**b**), HMBC- (**c**) spectra of apigenin 7-*O*-(4′′-*O*-*p*-*E*-coumaroyl)-glucoside (**4**).

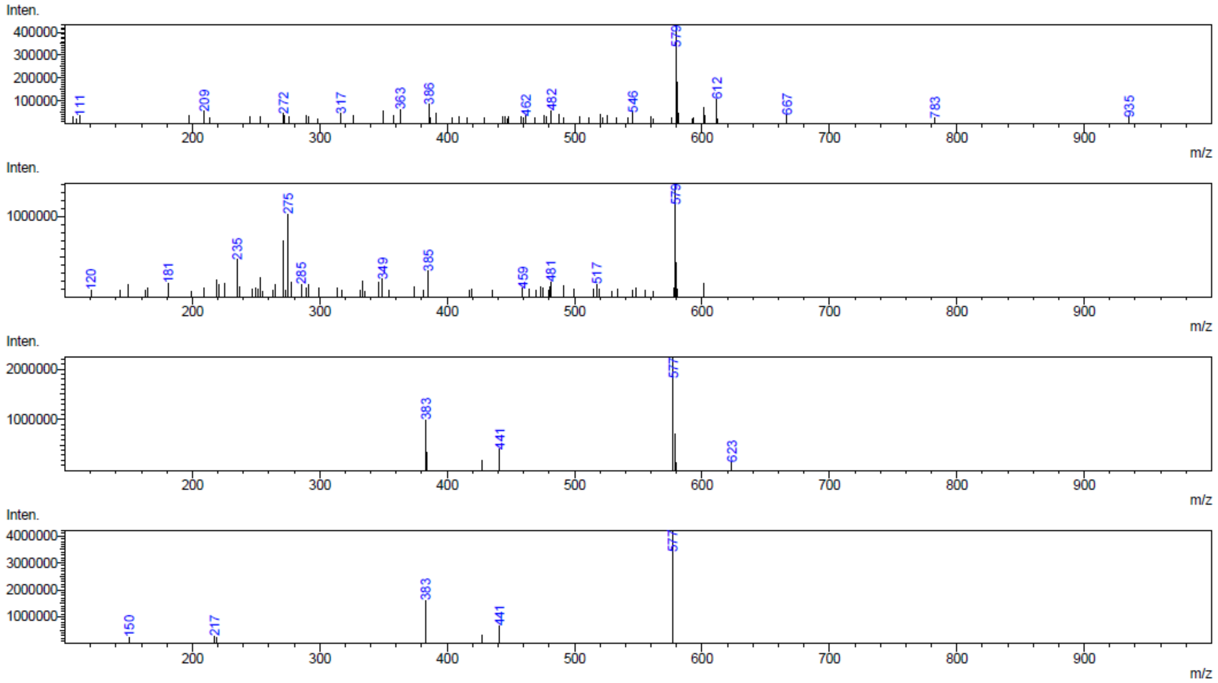

Fig. S7. ESI-MS spectra of apigenin 7-*O*-(4′′-*O*-*p*-*E*-coumaroyl)-glucoside (**4**). Spectra were recorded in positive ion mode (Q1+, Q3+) and negative ion mode (Q1-, Q3-).

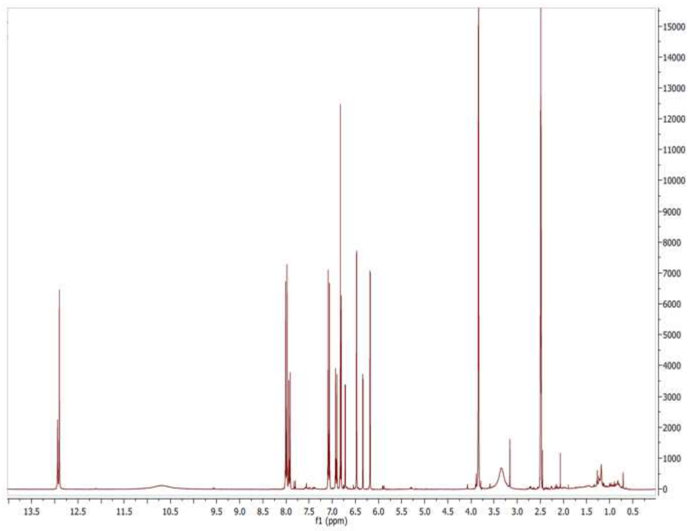

(a)

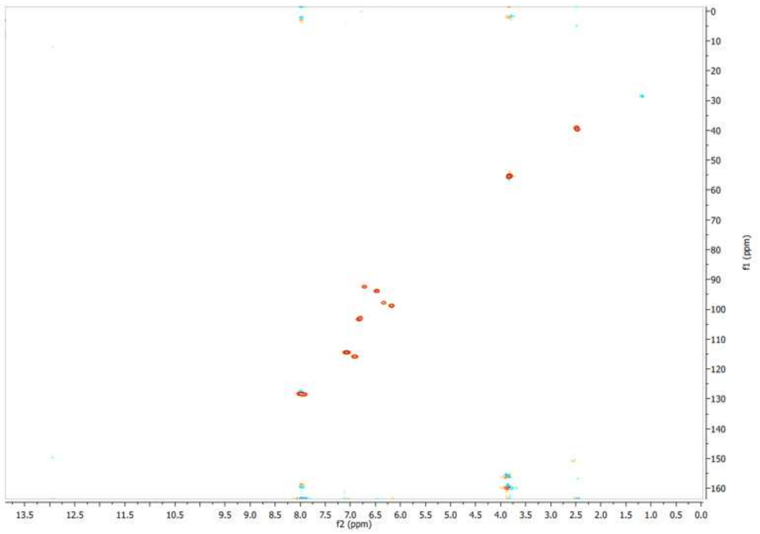

(b)

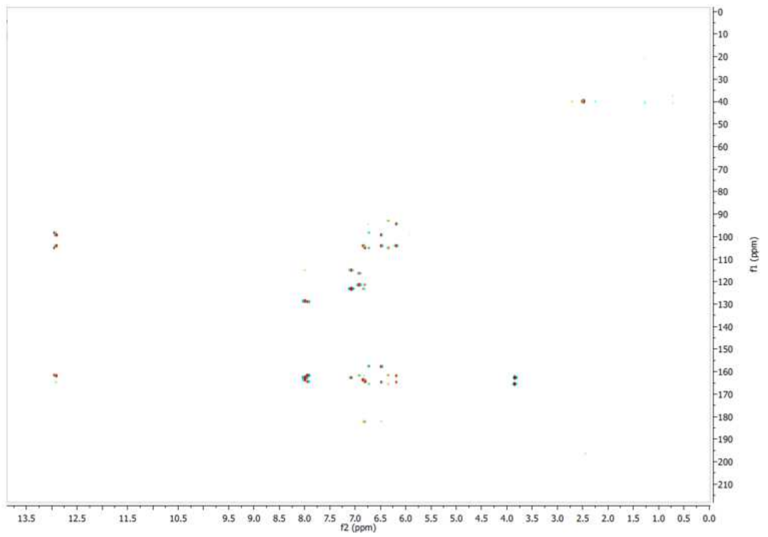

(c)

Fig. S8. ^{1}H-NMR- (**a**), HSQC- (**b**), HMBC- (**c**) spectra of acacetin and genkwanin (**5**+**6**).

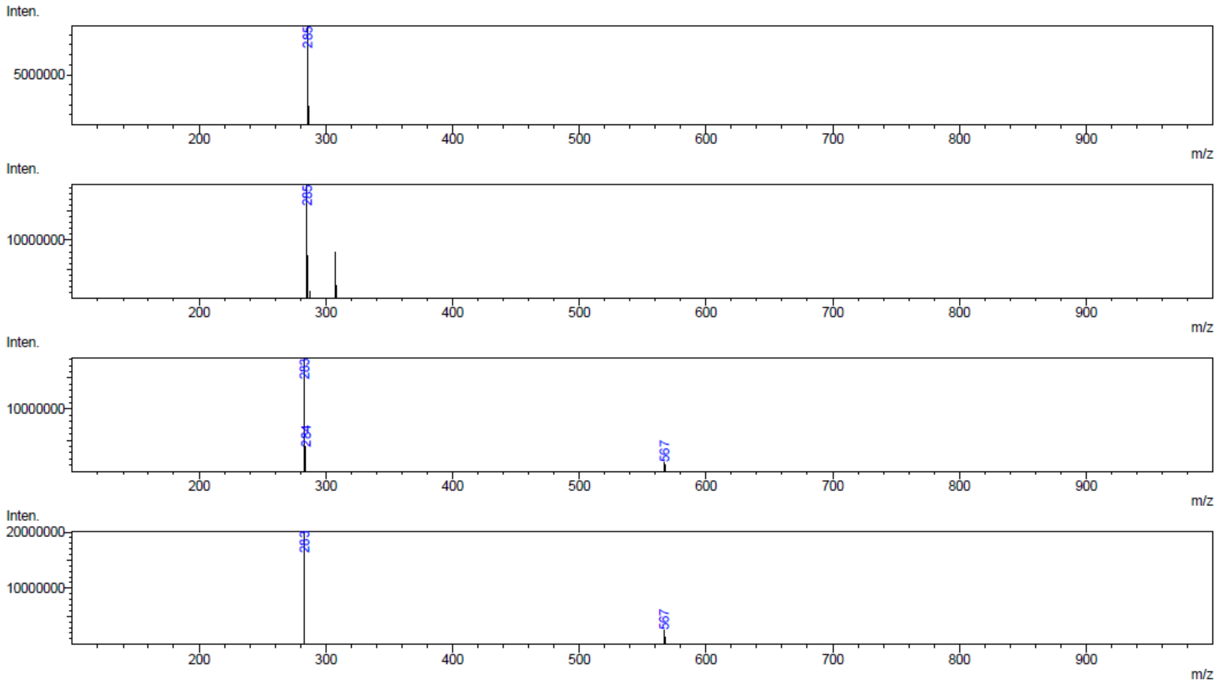

Fig. S9. ESI-MS spectra of acacetin and genkwanin (**5**+**6**). Spectra were recorded in positive ion mode (Q1+, Q3+) and negative ion mode (Q1-, Q3-).

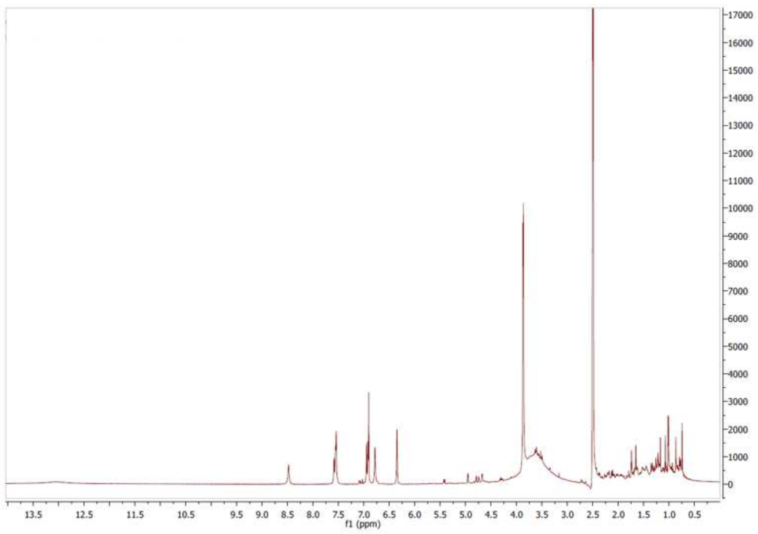

(a)

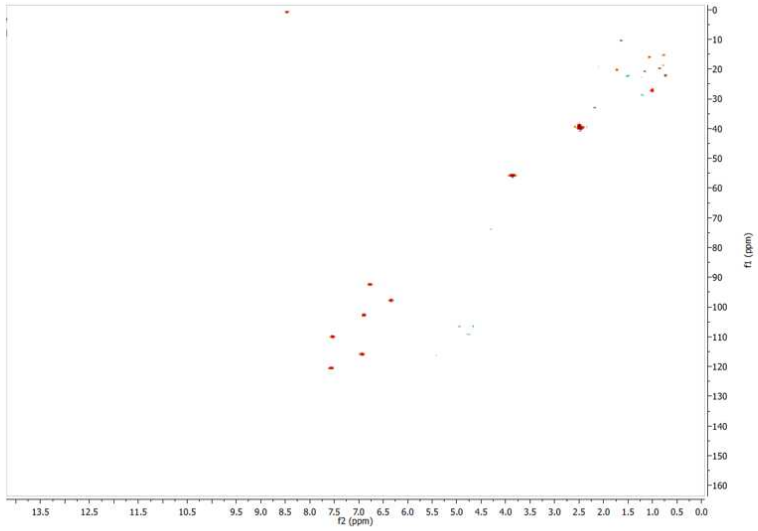

(b)

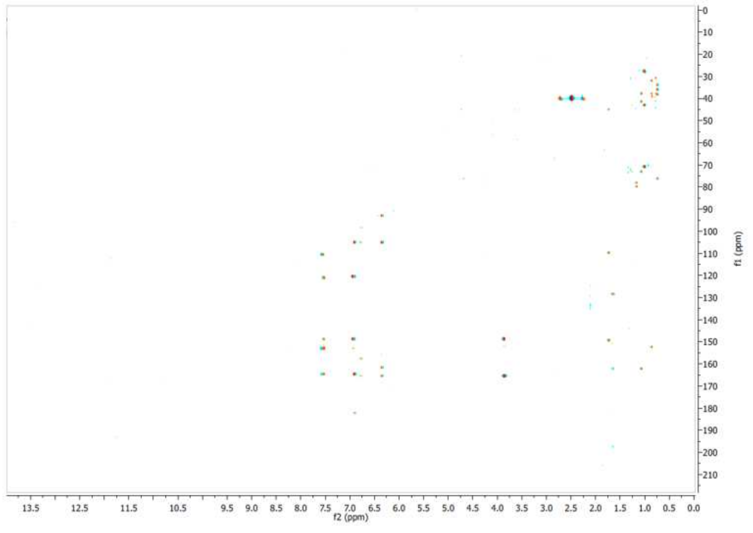

(c)

Fig. S10. ^{1}H-NMR- (**a**), HSQC- (**b**), HMBC- (**c**) spectra of velutin (**7**).

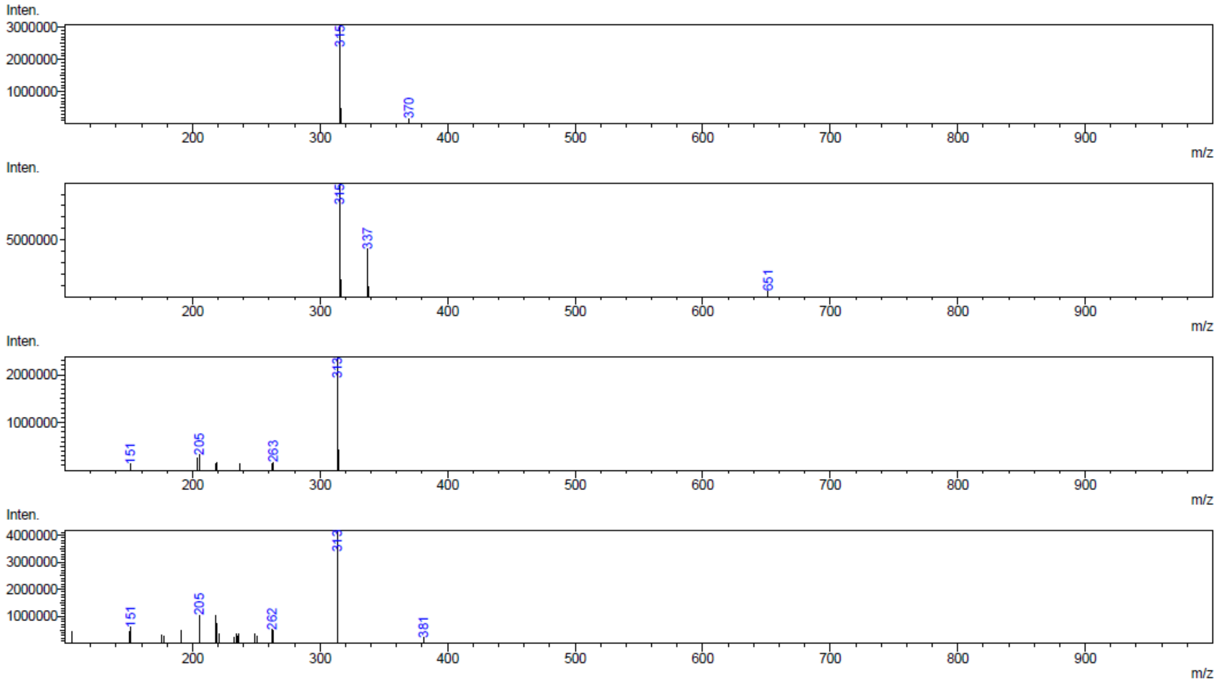

Fig. S11. ESI-MS spectra of velutin (**7**). Spectra were recorded in positive ion mode (Q1+, Q3+) and negative ion mode (Q1-, Q3-).

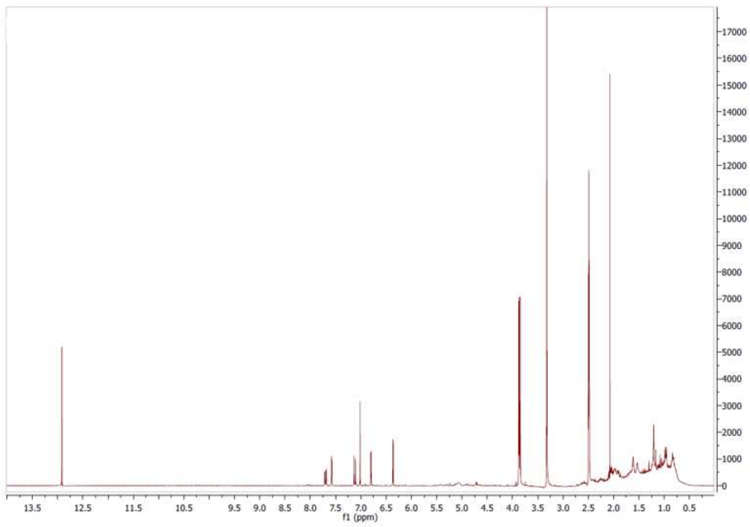

(a)

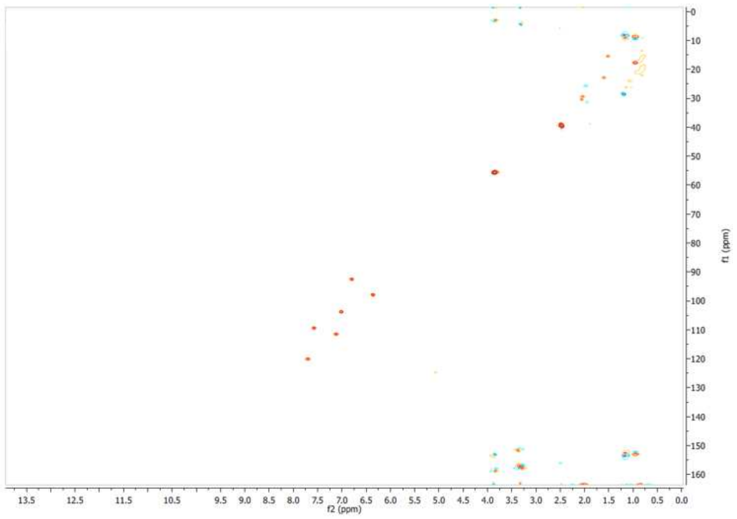

#

(b)

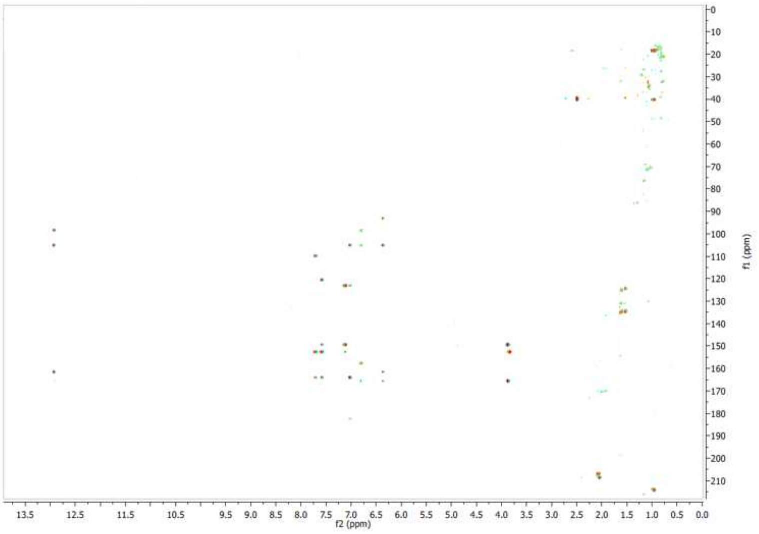

(c)

Fig. S12. ^{1}H-NMR- (**a**), HSQC- (**b**), HMBC- (**c**) spectra of gonzalitosin I (**8**).

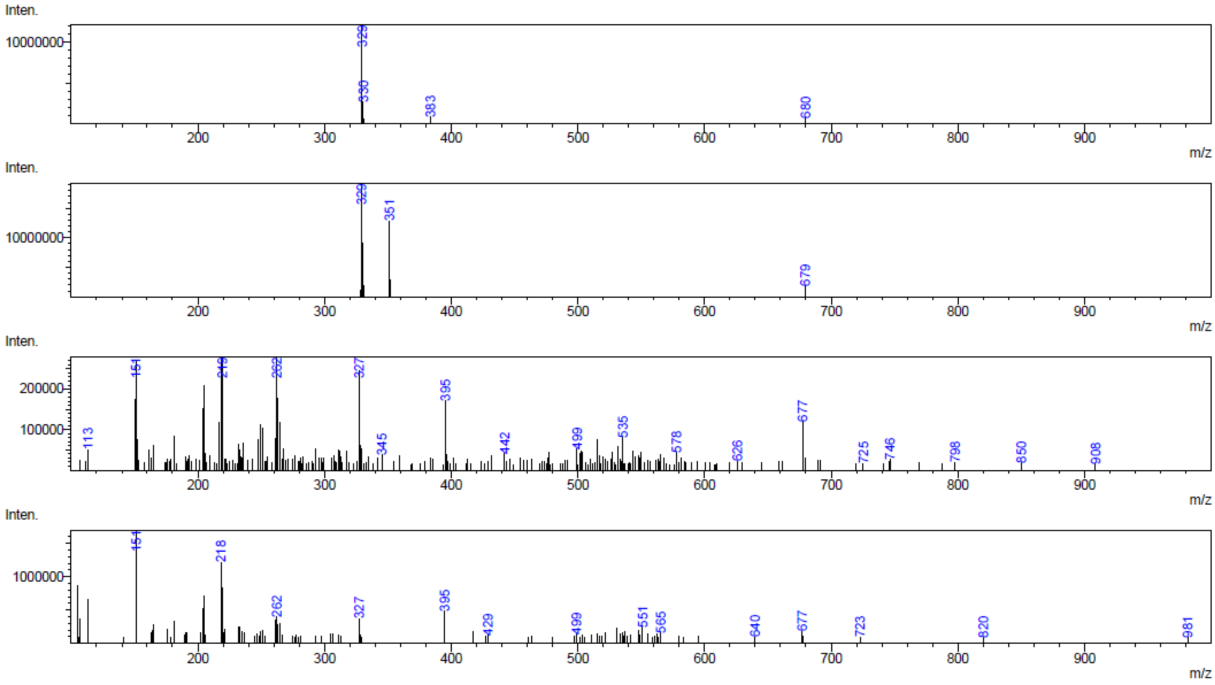

Fig. S13. ESI-MS spectra of gonzalitosin I (**8**). Spectra were recorded in positive ion mode (Q1+, Q3+) and negative ion mode (Q1-, Q3-).

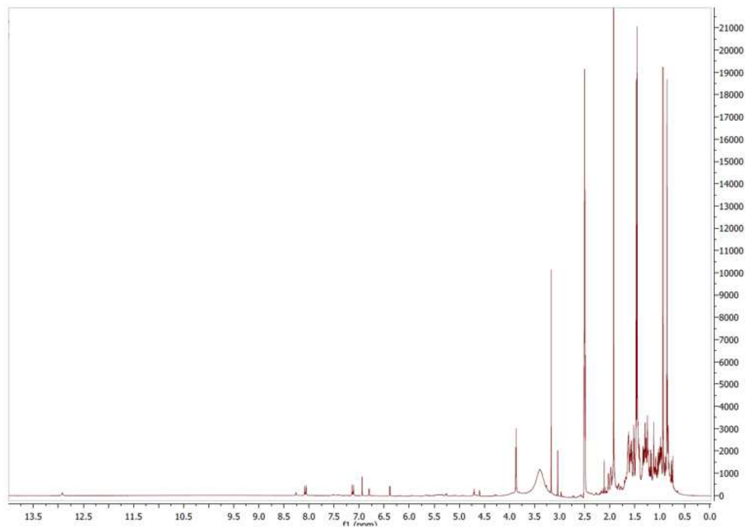

(a)

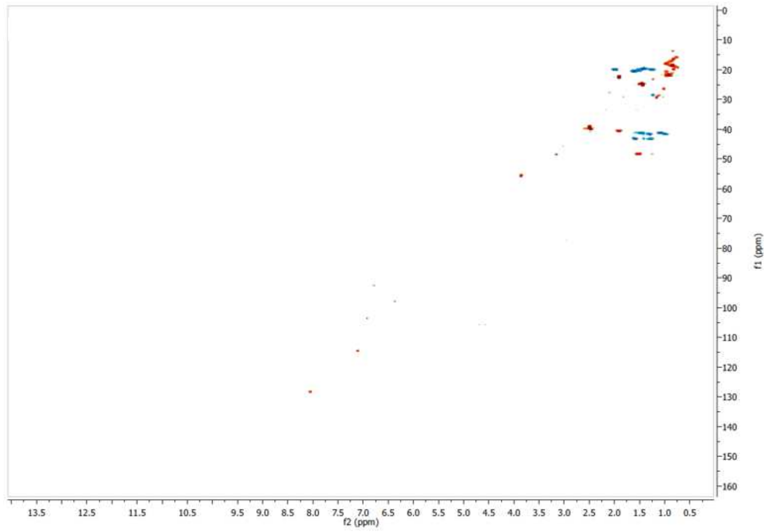

(b)

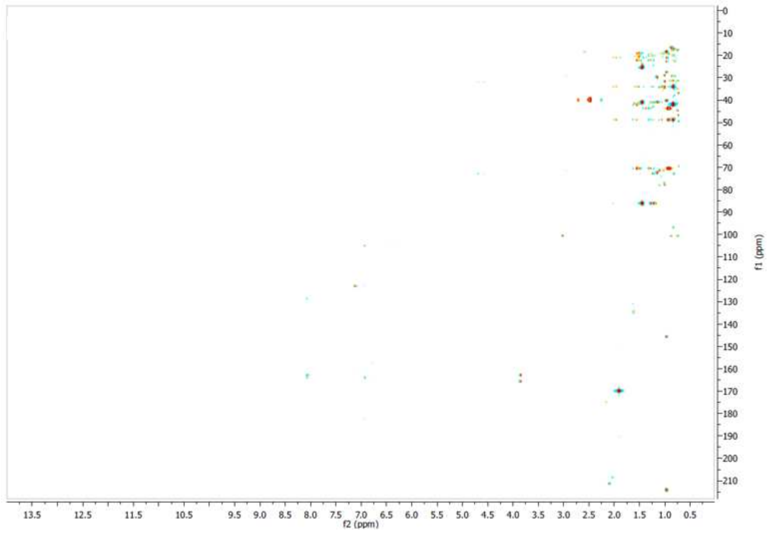

(c)

Fig. S14. ^{1}H-NMR- (**a**), HSQC- (**b**), HMBC- (**c**) spectra of acacetin 7-*O*-methyl ether (**9**).

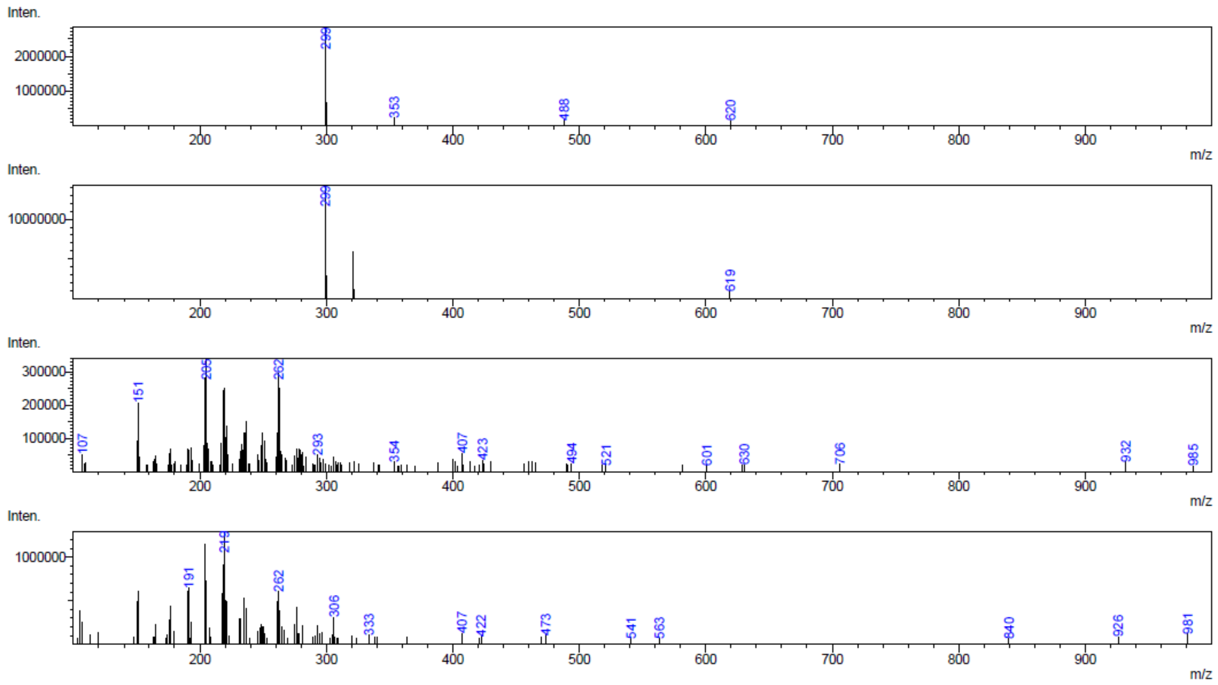

Fig. S15. ESI-MS spectra of acacetin 7-*O*-methyl ether (**9**). Spectra were recorded in positive ion mode (Q1+, Q3+) and negative ion mode (Q1-, Q3-).
